# Reported Use of Clinical Guidelines and Nursing Documentation Checklists Among Nurses in Kazakhstan: A Cross-Sectional Online Survey

**DOI:** 10.3390/healthcare14213780

**Published:** 2026-11-05

**Authors:** Bibinur Sydykova, Maira Sarsenbayeva, Zhanara Buribayeva, Aizhan Zholdasbekova, Gulnaz Kairatova, Laila Nazarova, Nazerke Narymbayeva, Meruyert Bolatova, Dariga Smailova, Nadira Aitambayeva, Shnara Svetlanova

**Affiliations:** 1 Department of Epidemiology, Evidence-Based Medicine and Biostatistics, Kazakhstan’s Medical University “KSPH”, Almaty 050060, Kazakhstan; bibinursk@gmail.com (B.S.);; 2 Medical College, Kazakhstan’s Medical University “KSPH”, Almaty 050060, Kazakhstan; 3 Department of Nursing, Asfendiyarov Kazakh National Medical University, Almaty 050012, Kazakhstan; zhanaraburibai@gmail.com; 4 Department of Healthcare Management, Kazakhstan’s Medical University “KSPH”, Almaty 050060, Kazakhstan; aizhanzhold@gmail.com (A.Z.); n.narymbay@gmail.com (N.N.); 5 Department of Public Health, Semey Medical University, Semey 071400, Kazakhstan; gulnaz.kairatova@smu.edu.kz; 6 Department of Nursing, Semey Medical University, Semey 071400, Kazakhstan; meruyert.bolatova@smu.edu.kz; 7 Data Center, Asfendiyarov Kazakh National Medical University, Almaty 050012, Kazakhstan; smailova.dariga@kaznmu.kz; 8 Department of Public Health and Healthcare, Kazakhstan’s Medical University “KSPH”, Almaty 050060, Kazakhstan; aitambaeva.nadira@gmail.com

**Keywords:** nursing practice, clinical guidelines, nursing documentation, evidence-based nursing, implementation, Kazakhstan

## Abstract

**Highlights:**

**What are the main findings?**

• Among 2263 nurses in a convenience sample in Kazakhstan, the most frequently reported clinical guidelines were those on pressure ulcer prevention (39.0%), patient- and family-centered care (37.1%), and fall prevention (34.3%); several disease-specific guidelines were reported by fewer than 17% of respondents (e.g., substance abuse, 13.0%; cerebral palsy, 14.2%; cardiac rehabilitation, 16.1%).
• Among nursing documentation checklists, the most frequently reported were allergic reaction care (40.3%) and preventing injuries and falls (34.5%); procedure- and setting-specific checklists were reported by fewer than 15% of respondents (e.g., care after a tracheostomy, 11.0%; gastric tube insertion in neonates and premature infants, 8.9%). All proportions use the whole sample (*N* = 2263) as the denominator.

**What are the implications of the main findings?**

• Reported use of guidelines and documentation tools varied widely and was low for many specialty- and procedure-specific items; whether this reflects limited implementation or limited applicability to respondents’ settings cannot be determined from these data.
• The causes of this variability were not examined; the role of nurse education, organizational support, and electronic documentation infrastructure in guideline and checklist use should be investigated in future research.

**Abstract:**

**Background/Objectives:** Evidence-based clinical guidelines and standardized nursing documentation are essential for healthcare quality and safety, yet evidence on their use by nurses in Kazakhstan is limited. This study assessed the reported use of clinical guidelines and nursing documentation checklists. **Methods:** A cross-sectional online survey with convenience sampling was conducted from December 2021 to February 2022; 2263 practicing nurses were analyzed. “Use” meant self-reported use in routine practice. Proportions with 95% confidence intervals were calculated for the whole sample. **Results:** Reported use varied across clinical areas. Pressure ulcer guidelines were used most often (39.0%; 95% CI 37.0–41.0), followed by patient- and family-centered care (37.1%), fall prevention (34.3%), and anti-smoking interventions (31.3%). Among documentation checklists, reported use was highest for allergic reaction care (40.3%) and preventing injuries and falls (34.5%) and below 15% for care after a tracheostomy, duodenal probing, and gastric tube insertion in neonates and premature infants, which apply only to specific patient groups. **Conclusions:** In this convenience sample, reported use of guidelines and checklists was low to moderate and uneven. The findings are not national implementation rates. The study design does not allow the causes of this variability to be identified; the role of education, organizational support, and electronic documentation should be examined in future research.

## 1. Introduction

Improving the quality, safety, and accessibility of healthcare services remains a global priority and is strongly supported by international healthcare organizations, including the World Health Organization [1]. Within modern healthcare systems, nurses play a central role in delivering patient-centered care and ensuring continuity of care through the application of evidence-based clinical practices. Nurse staffing and education have been recognized as important determinants of healthcare quality and clinical outcomes [2,3]. In response to increasing healthcare demands and the growing complexity of patient care, many countries have expanded advanced and specialized nursing roles, enabling nurses to take greater responsibility for care coordination, patient education, and clinical decision-making [4,5]. However, the effectiveness of these roles depends not only on professional education and competencies but also on supportive policies and organizational conditions [6].

Evidence-based clinical guidelines, standardized protocols, and structured nursing documentation are essential components of high-quality nursing practice [7]. These tools facilitate clinical decision-making, support interdisciplinary communication, and contribute to the delivery of consistent care [8]. Standardized nursing documentation is further associated with improved continuity of care and patient safety [9,10]. The successful implementation of clinical guidelines and documentation systems, however, requires adaptation to local healthcare contexts, adequate training, and institutional support. Clinical guidelines and documentation checklists represent different aspects of evidence-based practice. Clinical nursing guidelines are systematically developed recommendations that inform clinical decisions and care processes for specific conditions or risks, whereas standardized documentation checklists are structured forms used to record nursing assessments and interventions, typically at the initial nursing visit. Guideline use therefore reflects the application of evidence to care decisions, whereas checklist use reflects the structured recording of care; the two may diverge, and they were assessed separately in this study.

In Kazakhstan, nursing reform has been implemented through a series of legislative and organizational measures. The Code of the Republic of Kazakhstan “On the Health of the People and the Healthcare System” (No. 360-VI, 7 July 2020) [11] and the Rules for the Provision of Nursing Care (Order of the Minister of Health No. MoH RK-199/2020 of 23 November 2020, as amended) [12] established the current legal framework for nursing care, building on the Comprehensive Plan for the Development of Nursing until 2020 [13]. These measures were accompanied by the introduction of applied bachelor’s programs in nursing, the advanced practice nurse role, and national clinical nursing guidelines, standard operating procedures, and documentation forms [14,15]. Recent studies indicate, however, that the advanced practice model has not yet been adopted in all clinics [16,17]. Evidence on the extent to which clinical guidelines and standardized nursing documentation have been integrated into routine nursing practice remains limited.

Previous studies suggest that the availability of clinical guidelines alone does not guarantee their implementation in everyday clinical care. Systematic reviews identify gaps in awareness and knowledge, insufficient training, organizational barriers, limited resources, workload, and the complexity of guideline formats as factors that may reduce adherence to evidence-based recommendations [18,19]. Documentation burden and variability in documentation practice have likewise been reported [20]. Successful implementation appears to depend on continuous education, supportive organizational environments, and strategies that integrate guidelines into daily clinical workflows [18,19].

Although guideline implementation and nursing documentation have been extensively investigated in Europe, North America, and parts of Asia, published evidence from Central Asian countries is limited. In Kazakhstan, available studies have examined the advanced practice nurse role and independent nursing consultations [16,17], and a previous analysis of the present survey dataset assessed the utilization and standardization of nursing documentation, including 13 of the 29 documentation checklists and standard operating procedures [14]. The reported use of the national clinical nursing guidelines introduced as part of the reform and of the remaining 16 documentation checklists has not been investigated. This information is relevant because the reform relies on these tools to translate national standards into routine nursing care, and baseline data on their reported use are needed to plan and evaluate implementation. Therefore, this study aimed to describe the reported use of clinical nursing guidelines and of the 16 previously unreported documentation checklists among nurses in Kazakhstan, presenting proportions with confidence intervals and explicit denominators, and to identify areas where reported use is low. The findings may inform the design of future implementation research and of educational, organizational, and policy initiatives.

## 2. Materials and Methods

### 2.1. Study Design and Participants

This cross-sectional online survey was conducted in Kazakhstan between December 2021 and February 2022. The target population included practicing nurses employed in healthcare organizations in several regions of Kazakhstan. Eligible participants were nurses with vocational secondary medical education, higher nursing education, or an applied bachelor’s degree in nursing. Nurses who were not actively practicing at the time of the survey or who declined participation were excluded.

This study uses data from a multi-region convenience-sample online survey conducted in 2021–2022. A previous publication based on the same survey dataset reported participant characteristics, awareness of nursing reforms, 13 of the 29 documentation checklists of item 15 (limb edema, urinary catheter, obstetric hemorrhage, pulse oximetry, bronchial asthma, gastroenteritis and colitis, antibiotic sensitivity testing, COVID-19 care in children, acute respiratory infection/COVID-19 filter visit, nasogastric feeding, pressure ulcers, oxygen therapy, and MACS), and standard operating procedures [14]. The present article reports the other 16 checklists and the use of clinical nursing guidelines (item 24, not reported previously), analyzed by clinical area with confidence intervals and explicit denominators. Table 1 and the sampling frame overlap with the previous publication; this overlap is disclosed here and in Section 5.

Participants were recruited using convenience sampling through an official invitation letter of the Semey Medical University (NJSC “Medical University Semey”) dated 22 December 2021, addressed to the heads of primary health care organizations in Kazakhstan, which contained the survey link and asked them to facilitate the survey among their nurses. Because the invitation was addressed to heads of primary health care organizations, and dissemination depended on whether they forwarded the link, nurses working in primary health care were probably over-represented, and nurses in inpatient, intensive care, and neonatal settings were under-represented. Organizations more engaged with the nursing reform (for example, pilot sites for nursing development) and nurses more involved in documentation may also have been more likely to participate, which could result in higher reported use than in the wider nursing workforce.

Region of work was not asked directly. Respondents named their medical organization as free text (item 7) in a non-standardized form (e.g., abbreviations and facility numbers), so a region could be identified for only a minority of respondents; based on the list of participating organizations and the organization names given by respondents, respondents worked in several regions, including the cities of Almaty and Astana and the Akmola, Kostanay, Kyzylorda, Pavlodar, Turkistan, West Kazakhstan, and Zhambyl regions. The distribution of respondents by region is therefore not reported. Participation was voluntary and anonymous. Prior to completing the questionnaire, all participants received information regarding the purpose of the study and provided informed consent electronically.

A government policy document adopted in 2019 reported 175,705 mid-level medical personnel in Kazakhstan [21]. This workforce category is broader than nurses alone and should not be interpreted as the number of practicing nurses in 2021.

For the purposes of this study, “nurse” was defined as a specialist with vocational secondary medical education, higher nursing education, or an applied bachelor’s degree in nursing. The minimum sample size was calculated using Epi Info StatCalc (Centers for Disease Control and Prevention, Atlanta, GA, USA, assumed population size 175,000; expected proportion 50%; margin of error 5%; confidence level 99.99%; design effect 1.0), yielding a minimum of 1501 respondents. The 99.99% confidence level was chosen at the planning stage as a deliberately conservative setting to obtain a larger minimum sample because many items were to be estimated and the number of nurses who would participate could not be predicted; it was used only for sample size planning, and all estimates in this article are reported with 95% confidence intervals. The achieved sample (*n* = 2263) exceeded this requirement. This calculation is theoretical: it assumes simple random sampling, and because sampling was non-probabilistic, reaching the target sample size does not compensate for selection bias or establish national representativeness. Under simple random sampling assumptions, a sample of this size would provide an approximate margin of error of ±2.1 percentage points for a proportion of 50% at the 95% confidence level; this theoretical precision does not account for selection bias in the present convenience sample. Likewise, the 95% confidence intervals reported in this article reflect random sampling variability only and do not account for systematic error arising from the non-probabilistic sample.

Because the survey link was circulated through open channels, the number of nurses who received it could not be determined, and a conventional response rate cannot be calculated. For the same reason, non-response bias could not be assessed.

In total, 2276 questionnaires were submitted; 13 were excluded as invalid (non-numeric or implausible age or work-experience entries, nonsensical free-text entries, a contradictory answer on sex, or respondents who were not nurses), leaving 2263 for analysis. The proportion of submitted questionnaires retained for analysis was 99.4% (2263/2276).

### 2.2. The Tool and Data Collection Techniques

Data were collected using a structured self-administered questionnaire developed by the research team specifically for this study. The questionnaire was designed in accordance with the study objectives and covered key aspects of evidence-based nursing practice, including the use of clinical guidelines, application of standardized nursing documentation checklists, and selected demographic and professional characteristics of respondents.

The final questionnaire consisted of 27 items (Supplementary Files S1 and S2). Items 1–9 covered sociodemographic and professional characteristics, including position, work experience, qualification category, the name of the respondent’s medical organization (free text, item 7), and whether the organization is a pilot site for nursing development (item 8). Items 10–14, 16–23, and 25–26 covered awareness of and attitudes toward nursing reforms and documentation. Item 12 asked whether respondents keep nursing documentation, and item 13 asked whether they conduct nursing consultations with patients (yes/no).

Item 15 asked which of the 29 nursing documentation checklists respondents use at the initial nursing visit; item 24 asked which of the 15 clinical nursing guidelines they use in their work; and item 27 asked which of the 30 standard operating procedures they use. For items 15, 24, and 27, respondents ticked each tool they use (multiple responses allowed). Accordingly, “use” was defined as self-reported use by the respondent in routine practice (for checklists, at the initial nursing visit); the instrument did not distinguish availability from frequency or quality of use, and it did not include a “not applicable” option, so an unticked item may reflect either non-use or non-applicability. Table 1 presents sex, age, position, and qualification category. Education level and work experience were collected but are not shown because they were reported for the same sample previously [14]; item 7 (name of the medical organization) is summarized in the text of Section 3, and item 8 (pilot site) is not analyzed in this article. In addition, this article reports items 12–13, 16 of the 29 checklists of item 15, and item 24; the other items (awareness and attitudes, the remaining 13 checklists, and standard operating procedures) were reported previously [14]. Before distribution, the questionnaire was reviewed by members of the research team with expertise in nursing, public health, healthcare management, and evidence-based medicine to assess clarity, relevance, and consistency with the study objectives. The questionnaire was not intended to generate a psychometric scale; therefore, internal consistency testing was not performed, and individual items were analyzed descriptively. Prior to distribution, the questionnaire was pilot-tested among a small group of practicing nurses, whose feedback was used to improve the wording of several items; the number and characteristics of pilot participants were not formally recorded. The expert review and pilot test were intended solely to assess the clarity and readability of the items and did not constitute a formal validation of the instrument. Although the absence of internal consistency testing is consistent with the descriptive, item-level purpose of the questionnaire, the lack of formal validity and reliability assessment remains a limitation (Section 5).

Data were collected electronically using an online survey format (Google Forms, Google LLC, Mountain View, CA, USA). The survey link, together with information about the study and electronic informed consent, was distributed through the official invitation letter described above (Supplementary File S3). Completion of the questionnaire required approximately 20–25 min. All responses were anonymous. No personally identifiable information was collected. Prior to statistical analysis, submitted questionnaires were screened for validity and consistency; questionnaires with non-numeric or implausible age or work-experience entries, nonsensical free-text entries, contradictory answers on sex, or respondents who were not nurses were excluded from the final dataset.

### 2.3. Statistical Analysis

Statistical analyses were performed using IBM SPSS Statistics version 20.0 (IBM Corp., Armonk, NY, USA). Descriptive statistics were used to summarize the demographic and professional characteristics of the participants, as well as patterns of clinical guideline and nursing documentation checklist utilization. Categorical variables are presented as frequencies and percentages with 95% confidence intervals calculated using the Wilson score method; continuous variables are summarized using means, standard deviations (SDs), ranges, and 95% confidence intervals. Unless otherwise stated, the denominator for all proportions was the whole sample (*N* = 2263). Because several tools apply only to specific patient groups, whole-sample proportions represent the share of all respondents reporting use rather than utilization among nurses for whom each tool is clinically applicable. Specialty or department was not collected, and the questionnaire did not include a “not applicable” response option. Therefore, clinically applicable denominators could not be determined for procedure- and specialty-specific tools, and whole-sample denominators are reported for these items. Low proportions should not be interpreted as non-adherence, because an unselected item may indicate either non-use or non-applicability.

Work setting, specialty, and region were not analyzed as variables (see Section 5). Because the checklists refer to the initial nursing visit, checklist proportions were additionally calculated among respondents who reported conducting nursing consultations with patients (item 13 = “Yes”) (Supplementary Table S1). Only valid questionnaires were included in the final analysis. Results are presented as descriptive measures to provide an overview of the reported use of clinical guidelines and standardized nursing documentation among nurses in Kazakhstan.

The study is reported in accordance with the Strengthening the Reporting of Observational Studies in Epidemiology (STROBE) statement for cross-sectional studies; the completed checklist is provided in Supplementary File S4.

### 2.4. Ethical Considerations

The study protocol was reviewed and approved by the Ethics Committee of Semey Medical University (Approval No. 2, dated 28 October 2020). All procedures were conducted in accordance with ethical standards for research involving human participants.

## 3. Results

A total of 2263 nurses participated in the survey. Of the respondents, 3.6% were male, and 96.4% were female. The mean age of participants was 40.16 ± 12.38 years, with an age range from 18 to 66 years. Most respondents were general practice nurses (75.3%), while 2.8% were advanced practice nurses. Regarding professional qualification, the highest qualification category was reported by 44.0% of participants. Detailed demographic and professional characteristics of the study population are presented in Table 1.

**Table 1:** General characteristics of the study participants (*N* = 2263).

Variables		*n*	%
Sex	Male	82	3.6
	Female	2181	96.4
Age (years)	Mean ± SD	40.16 ± 12.38	
	Range (Min–Max)	18–66	
	95% CI	39.65–40.67	
Position	General practice nurse	1703	75.3
	Advanced practice nurse	63	2.8
	Specialized nurse	328	14.5
	Senior nurse	142	6.3
	Chief nurse	22	1.0
	Deputy Director of Nursing	4	0.2
	Director of a nursing care center	1	<0.1
Qualification	Higher	996	44.0
	First	314	13.9
	Second	188	8.3
	No category	765	33.8

SD, standard deviation; CI, confidence interval.

Respondents worked in medical organizations in several regions of Kazakhstan. Region and work setting were not recorded as structured variables (item 7 was a free-text field with non-standardized organization names), and specialty was not collected; the distribution of respondents by region, work setting, and specialty is therefore not reported, comparison with the national nursing workforce is not possible, and the sample should not be regarded as representative of it.

Reported use of clinical guidelines was moderate to low (Table 2). Nurses most frequently reported using guidelines related to the prevention and treatment of pressure sores (39.0%; 95% CI 37.0–41.0), followed by patient- and family-centered care, fall prevention, the continuum of care, and smoking cessation interventions (31.3–37.1%). Guidelines for chronic heart failure, cardiovascular risk assessment, asthma care, and psychosocial support of oncology patients were reported by 23.8–29.4% of respondents. Lower rates were observed for guidelines related to pediatric neurological conditions (cerebral palsy), substance abuse, and cardiac rehabilitation (13.0–16.1%). All proportions use the whole sample as the denominator.

**Table 2:** Reported use of clinical guidelines among participating nurses (*N* = 2263).

Clinical Guideline	Yes, *n* (% [95% CI])	Not Ticked, *n* (%)
Prevention and treatment of pressure sores	883 (39.0% [37.0–41.0])	1380 (61.0%)
Patient and family-centered care	839 (37.1% [35.1–39.1])	1424 (62.9%)
Application of anti-smoking interventions in daily nursing practice	708 (31.3% [29.4–33.2])	1555 (68.7%)
Caring for the adult patient with asthma: promoting asthma control	576 (25.5% [23.7–27.3])	1687 (74.5%)
Chronic heart failure: a clinical management guide for nurses (PCP and inpatient)	665 (29.4% [27.5–31.3])	1598 (70.6%)
Adapted clinical nursing guidelines for the management of children with cerebral palsy	321 (14.2% [12.8–15.7])	1942 (85.8%)
Rehabilitation interventions in stroke. Clinical nursing guidelines	456 (20.2% [18.5–21.9])	1807 (79.8%)
Prevention of falls and reduction in fall injuries. Adapted clinical care guidelines	776 (34.3% [32.4–36.3])	1487 (65.7%)
Management of chronic pain. Adapted clinical care guidelines	436 (19.3% [17.7–20.9])	1827 (80.7%)
Type 2 diabetes mellitus in adults. Adapted clinical nursing guidelines	486 (21.5% [19.8–23.2])	1777 (78.5%)
Cardiovascular risk assessment and prevention	610 (27.0% [25.2–28.8])	1653 (73.0%)
Screening, assessment and management of adult patients with malignancy associated with physiological distress and subsequent depression	538 (23.8% [22.1–25.6])	1725 (76.2%)
Adapted clinical nursing guidelines for substance abuse categories	294 (13.0% [11.7–14.4])	1969 (87.0%)
Provision of a continuum of care	718 (31.7% [29.8–33.7])	1545 (68.3%)
Cardiac rehabilitation	364 (16.1% [14.6–17.7])	1899 (83.9%)

Multiple responses were allowed; therefore, the sum of percentages exceeds 100%. Data are presented as *n* (%); 95% confidence intervals (Wilson score) are given in brackets. Denominator: *N* = 2263 (all respondents). “Not ticked” means that the respondent did not select the guideline; it does not indicate non-adherence or a clinical failure, and it may reflect either non-use or non-applicability to the respondent’s practice. The proportions should not be interpreted as implementation rates among nurses for whom each guideline is clinically applicable.

The use of standardized nursing documentation checklists during the initial nursing visit varied across clinical areas (Table 3). The highest reported use was observed for allergic reactions care (40.3%; 95% CI 38.3–42.3), preventing injuries and falls (34.5%), and monitoring a patient with acute stroke (27.5%). Lower rates were reported for checklists related to diabetes management (13.6–23.2%), care of a venous catheter (19.5%), nursing education for patients with CHF (19.4%), monitoring a glaucoma patient (14.2%), and care after a tracheostomy (11.0%). Because several of these tools apply only to specific patient groups, whole-sample proportions do not estimate utilization among nurses for whom each tool is clinically applicable and should not be interpreted as evidence of non-adherence. Of the 2263 respondents, 2097 (92.7%) reported keeping nursing documentation (item 12), and 1715 (75.8%) reported conducting nursing consultations with patients (item 13) [14]. Among respondents who conduct nursing consultations (*n* = 1715), reported use of the 16 checklists in Table 3 and Table 4 ranged from 8.5% (gastric tube insertion in neonates and premature infants) to 41.2% (allergic reactions care) (Supplementary Table S1), compared with 8.6–40.3% in the whole sample; restricting the denominator therefore did not change the overall pattern.

**Table 3:** Utilization of standardized nursing documentation checklists during the initial nursing Visit (*N* = 2263).

ICD-10 Code and Category	Checklist	Yes, *n* (% [95% CI])	Not Ticked, *n* (%)
I61–I64 (Intracerebral hemorrhage; cerebral infarction; stroke, not specified as hemorrhage or infarction)	Monitoring a patient with acute stroke	622 (27.5% [25.7–29.4])	1641 (72.5%)
H40 (Glaucoma)	Monitoring a glaucoma patient	322 (14.2% [12.9–15.7])	1941 (85.8%)
T78.2, T78.4 (Anaphylactic shock, unspecified; allergy, unspecified)	Allergic reactions care	912 (40.3% [38.3–42.3])	1351 (59.7%)
S00–T14 (Injuries to specific and unspecified body regions)	Care of a patient with fractures/injuries	518 (22.9% [21.2–24.7])	1745 (77.1%)
W00–W19 (Falls, stumbles and other mechanical injuries caused by external causes)	Preventing injuries and falls	781 (34.5% [32.6–36.5])	1482 (65.5%)
E11 (Type 2 diabetes mellitus)	Diabetes mellitus in adults	525 (23.2% [21.5–25.0])	1738 (76.8%)
E10 (Type 1 diabetes mellitus)	Diabetes mellitus in children and adolescents	308 (13.6% [12.3–15.1])	1955 (86.4%)
Z45.2 (Adjustment and management of vascular access device)	Care of a venous catheter	441 (19.5% [17.9–21.2])	1822 (80.5%)
I50 (Heart failure)	Nursing education for patients with CHF	440 (19.4% [17.9–21.1])	1823 (80.6%)
Z43.0 (Attention to tracheostomy)	Care after a tracheostomy	248 (11.0% [9.7–12.3])	2015 (89.0%)

Note: Multiple responses were allowed; therefore, the sum of percentages exceeds 100%. Data are presented as *n* (%); 95% confidence intervals (Wilson score) are given in brackets. Denominator: *N* = 2263 (all respondents). “Not ticked” means that the respondent did not select the checklist; it does not indicate non-adherence or a clinical failure, and it may reflect either non-use or non-applicability to the respondent’s practice. The proportions should not be interpreted as implementation rates among nurses for whom each checklist is clinically applicable; this applies particularly to setting-specific tools such as care after a tracheostomy. All codes in this table are ICD-10 codes and indicate the clinical area to which each checklist relates: I61–I64, H40, T78.2/T78.4, S00–T14, E10, E11, and I50 are diagnosis codes; W00–W19 are external-cause codes; and Z45.2 and Z43.0 are Z codes for contact with health services, not diagnoses. ICD-10 does not encode age; E10 is used for the checklist for children and adolescents, in whom type 1 diabetes predominates, and the age group is defined by the checklist.

The use of additional nursing documentation checklists was generally low (Table 4). The highest reported use was for the checklist on nurse action when identifying a patient with an infectious disease on the ward (30.2%; 95% CI 28.3–32.1). Lower rates were observed for tools addressing determination of daily diuresis and water balance in chronic heart failure (14.6%), administering medication using a perfuser (14.5%), recognizing white coat hypertension (12.6%), gastric tube insertion in neonates and premature infants (8.9%), and duodenal probing (8.6%). As these procedures are performed mainly in inpatient and neonatal settings, the whole-sample proportions do not indicate how often the checklists are used where they apply.

**Table 4:** Additional nursing documentation checklists used during the initial nursing visit (*N* = 2263).

ICD-10 Code and Category, or ICD-9-CM Procedure Code	Checklist	Yes, *n* (% [95% CI])	Not Ticked, *n* (%)
R03.0 (Elevated blood-pressure reading, without diagnosis of hypertension)	Recognizing white coat hypertension	286 (12.6% [11.3–14.1])	1977 (87.4%)
I50 (Heart failure)	Determination of daily diuresis and water balance in chronic heart failure	330 (14.6% [13.2–16.1])	1933 (85.4%)
A00–B99 (Some infectious and parasitic diseases)	Nurse action when identifying a patient with an infectious disease on the ward	683 (30.2% [28.3–32.1])	1580 (69.8%)
Z45.1 (Adjustment and management of infusion pump)	Administering medication using a perfuser	329 (14.5% [13.1–16.1])	1934 (85.5%)
ICD-9-CM 96.08 (Insertion of (naso-)intestinal tube)	Carrying out duodenal probing	194 (8.6% [7.5–9.8])	2069 (91.4%)
ICD-9-CM 96.07 (Insertion of other (naso-)gastric tube)	Gastric tube insertion in neonates and premature infants	202 (8.9% [7.8–10.2])	2061 (91.1%)

Note: Multiple responses were allowed; therefore, the sum of percentages exceeds 100%. Data are presented as *n* (%); 95% confidence intervals (Wilson score) are given in brackets. Denominator: *N* = 2263 (all respondents). “Not ticked” means that the respondent did not select the checklist; it does not indicate non-adherence or a clinical failure, and it may reflect either non-use or non-applicability. Several of these checklists apply only to specific care settings (e.g., gastric tube insertion in neonates and premature infants, duodenal probing, administering medication using a perfuser); because the whole sample is used as the denominator, their proportions should be interpreted with particular caution and not as implementation rates among eligible nurses. R03.0, I50, and A00–B99 are ICD-10 diagnosis codes (R03.0 includes white-coat hypertension); Z45.1 is an ICD-10 Z code for contact with health services; and ICD-9-CM 96.08 and 96.07 are procedure codes (96.07 covers insertion of nasogastric and orogastric tubes and does not identify the neonatal population).

Item-level data on assessment tools used in the care of children with cerebral palsy, post-stroke rehabilitation practices, and communication-related algorithms from the same survey have been reported previously [14] and are not repeated here.

## 4. Discussion

The present study assessed the reported use of clinical guidelines and standardized nursing documentation checklists among nurses in Kazakhstan. The findings indicate that reported use varied widely across clinical areas and was generally low to moderate. The highest reported use concerned basic patient-safety tools (pressure ulcer prevention, fall prevention, allergic reaction care, and infection control), whereas disease-specific and procedure-related tools were reported less often. Because many of these tools apply only to specific settings and the whole sample was used as the denominator, low proportions for such items cannot be interpreted as non-adherence.

The observed variability in guideline utilization is consistent with international evidence that the availability of evidence-based recommendations does not necessarily translate into routine use [18,19,22]. Systematic reviews of nurses’ guideline use identify barriers such as limited awareness, knowledge and training, workload, complex guideline formats, and weak organizational support [18,19]. These barriers were not assessed in the present survey, so the extent to which they apply to our respondents is unknown.

The relatively higher reported use of guidelines on pressure ulcer prevention, fall prevention, and patient-centered care may reflect their long-standing role in safety-related practice; however, we did not collect data on curricula, accreditation, or institutional policies, so this explanation remains a hypothesis.

Reported checklist use varied across clinical areas, but these proportions should not be interpreted as measures of documentation quality or adherence among clinically relevant nurses. Recent evidence provides context for understanding documentation practices. A systematic review identified audit and feedback, education, and changes to documentation forms or systems as potential improvement strategies, although the certainty of evidence was very low [10]. A cross-sectional study in Ethiopian public hospitals found an association between in-service training and documentation practice [23]. Documentation burden and its association with clinician burnout have also been reported [20], while a workflow redesign project examined opportunities to streamline nursing documentation in electronic health records [24]. These studies assessed different settings and outcomes and therefore do not support direct comparisons with our utilization proportions. They identify potential organizational and workflow factors for future investigation in Kazakhstan; these factors were not measured in the present survey.

Several explanations other than limited implementation may account for the observed variability. First, the clinical applicability of the tools differs by specialty: checklists for care after a tracheostomy, duodenal probing, gastric tube insertion in neonates and premature infants, or administering medication using a perfuser are relevant mainly in inpatient, intensive care, or neonatal settings, whereas tools for pressure ulcers, falls, and allergic reactions apply to most patients. Second, the invitation was addressed to primary health care organizations, so the sample probably included relatively few nurses from settings where the specialized tools are used. Third, the availability of individual guidelines and checklists, in printed or electronic form, may have differed between organizations, and some tools may not yet have been introduced in all facilities at the time of data collection. Fourth, respondents may have interpreted “use” of a tool differently. Because specialty, work setting, and tool availability were not recorded, the relative contribution of these explanations cannot be determined.

The present study contributes to the limited body of evidence on evidence-based nursing practice in Central Asia. While nursing modernization and healthcare reforms in Kazakhstan have promoted the introduction of clinical guidelines and standardized documentation, the findings show that the reported use of these tools was uneven across areas of practice in this sample. Because implementation barriers were not assessed, these data cannot show whether this variability reflects a gap between policy-level reforms and routine nursing care or the alternative explanations discussed above. The findings provide descriptive baseline data that may inform the design of future implementation research in Kazakhstan and other countries undergoing healthcare system transformation.

Data were collected between December 2021 and February 2022, during the COVID-19 pandemic and shortly after the introduction of the current nursing-care regulations [12]; they therefore describe reported use only at that time, during the early implementation period, and do not allow conclusions about whether use has since increased or decreased. The following national developments are presented solely to place the study in context and to justify further field research; they do not provide evidence of the current level of guideline or checklist use. First, the 2020 Rules were amended in 2025 (Order No. 26 of 31 March 2025, registered with the Ministry of Justice No. 35890) [12]. Second, official data for 2025 indicate that more than 20,000 nurses with applied bachelor’s diplomas had been employed in health facilities over the preceding six years (a period that partly precedes and partly follows the data collection), representing approximately 14% of the 146,000 nurses practicing in 2025 [25], while nursing and midwifery density has remained stable at 93–100 per 10,000 population through 2024 [15]. Third, recent qualitative and patient-satisfaction studies indicate that the advanced practice model has not yet been adopted in all clinics [16,17]. Fourth, the 2025–2030 Central Asian Strategic Directions for Nursing and Midwifery, adopted by WHO/Europe and the ministries of health of five Central Asian countries in December 2025, identify standardized education and expanded professional roles as regional priorities [26]. Utilization may have changed since data collection; the present results should therefore be read as a historical baseline against which later studies can be compared, not as an estimate of current practice.

Although the present study cannot identify the reasons for the observed variability, it raises questions for future research. International studies have linked guideline and documentation use to continuous professional education, organizational support, and the integration of tools into clinical workflows and electronic documentation systems [10,18,19,20,23,24]. None of these factors was measured in the present survey; they should be regarded as working hypotheses rather than established causes, to be tested in Kazakhstan in studies that record specialty, work setting, tool availability, and training, and that verify use through documentation audit.

## 5. Strengths and Limitations

This study has several strengths. To our knowledge, it is one of the largest surveys examining the use of clinical guidelines and nursing documentation among nurses in Kazakhstan, although participants were recruited by convenience sampling. The large sample size and inclusion of nurses with diverse educational backgrounds, professional roles, and qualification levels provide a broad overview of reported practice among respondents.

Several limitations should also be acknowledged. First, the cross-sectional design does not allow causal relationships to be established. Second, guideline and checklist use was self-reported and was not verified against nursing records or by observation; responses may therefore be affected by recall bias and by social desirability bias, which would tend to overestimate use, particularly because the survey was distributed through employers’ official channels. Third, participants were recruited by convenience sampling through heads of primary health care organizations using an online survey, which may have introduced selection bias: nurses in primary health care, in organizations more engaged with the nursing reform, and nurses more interested in documentation may be over-represented. Because the number of nurses who received the invitation is unknown, a response rate could not be calculated, and non-response bias could not be assessed. The sample size calculation and the confidence intervals assume random sampling and reflect sampling variability only; they do not correct for these biases, and the results should not be interpreted as national or epidemiological estimates. Fourth, region and work setting were not recorded as structured variables, and specialty was not collected. Consequently, the distribution of the sample could not be described or compared with the national nursing workforce, and it was not possible to determine for which respondents a given guideline or checklist was clinically applicable. Fifth, the questionnaire had no “not applicable” option, so an unticked item cannot be classified as non-use or non-applicability; whole-sample proportions therefore probably underestimate use among nurses for whom a tool is relevant, especially for specialty- and procedure-specific tools, and the additional analysis among nurses who conduct nursing consultations only partly addresses this. Sixth, “use” did not distinguish availability from frequency or quality of use. Seventh, the questionnaire was developed for this study; expert review and pilot testing addressed only the clarity and readability of the items, and no formal assessment of validity or reliability was performed, so measurement error cannot be excluded. Eighth, data were collected in December 2021–February 2022, during the COVID-19 pandemic, and reflect reported use at that time only; they do not allow conclusions about subsequent trends. Finally, part of the dataset has been used in a previous publication [14], and only 16 of the 29 checklists of item 15 are reported here. The present study was primarily descriptive and did not explore predictors of guideline utilization; future studies should investigate demographic, educational, and organizational factors associated with implementation.

## 6. Conclusions

In this convenience sample of nurses in Kazakhstan, self-reported use of clinical guidelines and standardized nursing documentation checklists was low to moderate and varied across areas of nursing practice. Guidelines and checklists related to patient safety were reported more frequently, whereas disease-specific and procedure-oriented tools were reported less frequently; because many of these tools apply only to specific settings, the figures should not be interpreted as national implementation rates. The study design does not allow the causes of this variability to be identified, and the data should not be interpreted as epidemiological or national-level estimates. Future research should examine organizational, educational, and individual factors associated with guideline and checklist use among nurses in Kazakhstan, including the role of training, leadership engagement, and electronic documentation systems, using samples with recorded specialty and work setting and objective measures of use.

## Data Availability

The data presented in this study are available upon request from the corresponding author. The data are not publicly available due to privacy and ethical restrictions.

## Supplementary Materials

The following supporting information can be downloaded at https://www.mdpi.com/article/10.3390/healthcare14213780/s1, File S1: Questionnaire (English translation); File S2: Questionnaire (original-language version); File S3: Official invitation letter of the Semey Medical University (original, Russian); Table S1: Reported use of the 16 checklists among respondents who conduct nursing consultations (item 13 = “Yes”); File S4: STROBE checklist for cross-sectional studies.
