# Supplementary material for: Reported Use of Clinical Guidelines and Nursing Documentation Checklists Among Nurses in Kazakhstan: A Cross-Sectional Online Survey
Source: Healthcare (Basel). 2026 Nov 5;14(21):3780. doi: 10.3390/healthcare14213780 (PMC13763343; doi:10.3390/healthcare14213780)
Supplement: Supplementary file 1 [file healthcare-14-03780-s001.zip › supp m/File S4_STROBE Statement.pdf]

Supplementary File S4. STROBE Statement—Checklist of items that should be included in reports of cross-sectional studies

Reported Use of Clinical Guidelines and Nursing Documentation Checklists among Nurses in Kazakhstan: A Cross-Sectional Online Survey

Section/Topic	Item	Recommendation	Where reported in the manuscript
Title and abstract	1	(a) Indicate the study's design with a commonly used term in the title or the abstract; (b) provide an informative and balanced summary of what was done and what was found	Title; Abstract
Background/rationale	2	Explain the scientific background and rationale for the investigation being reported	Introduction, paragraphs 1–5
Objectives	3	State specific objectives, including any prespecified hypotheses	Introduction, final paragraph
Study design	4	Present key elements of study design early in the paper	Section 2.1
Setting	5	Describe the setting, locations, and relevant dates, including periods of recruitment, exposure, follow-up, and data collection	Section 2.1 (December 2021–February 2022; invitation letter of 22 December 2021; regions); Section 2.2
Participants	6	(a) Give the eligibility criteria, and the sources and methods of selection of participants	Section 2.1 (eligibility criteria; convenience sampling through heads of primary health care organizations)
Variables	7	Clearly define all outcomes, exposures, predictors, potential confounders, and effect modifiers	Section 2.2 (items 12, 13, 15, and 24; definition of "use")
Data sources/measurement	8	For each variable of interest, give sources of data and details of methods of assessment	Section 2.2 (self-administered online questionnaire, Files S1–S2; expert review and pilot test)
Bias	9	Describe any efforts to address potential sources of bias	Sections 2.1–2.3 (validity screening; restricted-denominator analysis, Table S1); Section 5
Study size	10	Explain how the study size was arrived at	Section 2.1 (sample size calculation; achieved n = 2,263)
Quantitative variables	11	Explain how quantitative variables were handled in the analyses	Section 2.3
Statistical methods	12	(a) Describe all statistical methods; (b) methods used to examine subgroups; (c) how missing data were addressed; (d) analytical	(a) Section 2.3; (b) Section 2.3, Table S1; (c) Sections 2.1–2.2 (invalid questionnaires excluded; tick-box items have no

		methods taking account of sampling strategy; (e) sensitivity analyses	missing values); (d) not applicable (non-probability sample), see Sections 2.1 and 5; (e) Section 2.3, Table S1
Participants	13	(a) Report numbers of individuals at each stage of study; (b) give reasons for non-participation; (c) consider use of a flow diagram	(a) Section 2.1 (2,276 submitted, 13 excluded, 2,263 analyzed; number invited unknown); (b) Section 2.1; (c) not provided (single-stage online survey; numbers given in text)
Descriptive data	14	(a) Give characteristics of study participants; (b) indicate number of participants with missing data for each variable of interest	(a) Results, Table 1; (b) not applicable (no missing values in analyzed variables)
Outcome data	15	Report numbers of outcome events or summary measures	Results, Tables 2–4
Main results	16	(a) Give unadjusted estimates and their precision (e.g., 95% CI); (b) report category boundaries; (c) translate relative risk into absolute risk	(a) Tables 2–4 (n, %, 95% CI); (b), (c) not applicable
Other analyses	17	Report other analyses done (subgroups, interactions, sensitivity analyses)	Results; Table S1
Key results	18	Summarise key results with reference to study objectives	Discussion, paragraph 1
Limitations	19	Discuss limitations of the study, taking into account sources of potential bias or imprecision	Section 5
Interpretation	20	Give a cautious overall interpretation of results	Discussion; Conclusions
Generalisability	21	Discuss the generalisability (external validity) of the study results	Section 5; Discussion
Funding	22	Give the source of funding and the role of the funders	Funding statement (no external funding)

Section numbers refer to the revised manuscript. Checklist items are abbreviated from the STROBE statement (von Elm E. et al., 2007; www.strobe-statement.org).
