# Supplementary material for: Reported Use of Clinical Guidelines and Nursing Documentation Checklists Among Nurses in Kazakhstan: A Cross-Sectional Online Survey
Source: Healthcare (Basel). 2026 Nov 5;14(21):3780. doi: 10.3390/healthcare14213780 (PMC13763343; doi:10.3390/healthcare14213780)
Supplement: Supplementary file 1 [file healthcare-14-03780-s001.zip › supp m/File_S1_Questionnaire_English_translation.pdf]

Supplementary File S1

Questionnaire (English translation of the original Russian/Kazakh version, File S2)

Translation of the Russian-language text of the bilingual (Russian/Kazakh) online questionnaire (Google Forms). Check-box items allow one answer unless "select all that apply" is stated. Item numbers correspond to File S2.

SURVEY

Dear participant! This survey is conducted as part of a study on improving the nursing service in the Republic of Kazakhstan by developing and implementing nursing documentation in healthcare organizations. The survey is anonymous, and its results will be used only in aggregated form. Thank you in advance for participating!

1. Age

2. Indicate your sex

- ☐ Male
- ☐ Female

3. Education

- ☐ Technical and vocational education in the specialty "Nursing"
- ☐ Applied bachelor's degree in "Nursing"
- ☐ Academic bachelor's degree in "Nursing"
- ☐ Master's degree in "Nursing"
- ☐ PhD in "Nursing"

4. Position

- ☐ General practice nurse
- ☐ Advanced practice nurse
- ☐ Specialized nurse
- ☐ Senior nurse
- ☐ Chief nurse
- ☐ Deputy director for nursing
- ☐ Director of a nursing care center

5. Work experience (years): ________

6. Qualification category

- ☐ Highest
- ☐ First
- ☐ Second
- ☐ No category

7. In which medical organization do you work? (free text) ________

8. Is your organization a pilot site for the development of the nursing service?

- ☐ Yes
- ☐ No

8.1 Source: Order of 4 July 2018 No. 419 "On the implementation of a pilot project on the introduction of a new model of the nursing service in healthcare organizations" (reference link provided in the original).

9. Describe your job functions (free text) ________

10. Do you know that the position of “deputy head for nursing” has been introduced in Kazakhstan?

☐ Yes
☐ No

11. Do you know that the position of “advanced practice nurse” has been introduced in Kazakhstan?

☐ Yes
☐ No

12. Do you keep nursing documentation?

☐ Yes
☐ No

13. Do you conduct nursing consultations with patients?

☐ Yes
☐ No

14. If yes, what data do you collect at the initial nursing visit? (free text) ________

15. Which nursing documentation (checklists) do you use at the initial nursing visit? (select all that apply)

☐ Monitoring of a patient with acute cerebrovascular accident
☐ Monitoring of a patient with glaucoma
☐ Assistance in an allergic reaction
☐ Assistance to a patient with fractures/injuries
☐ Prevention of injuries and falls
☐ Diabetes mellitus in adults
☐ Diabetes mellitus in children and adolescents
☐ Care of a venous catheter
☐ Teaching a patient with chronic heart failure (CHF) self-care
☐ Care after tracheostomy
☐ Recognition of white-coat hypertension
☐ Determination of daily diuresis and water balance in chronic heart failure
☐ Actions of the nurse when a patient with an infectious disease is identified on the ward
☐ Assessment of hand function using the Manual Ability Classification System (MACS) in children with cerebral palsy
☐ Administration of medicines using a perfusor (infusion pump)
☐ Duodenal probing (intubation)
☐ Insertion of a gastric tube in newborn and premature infants
☐ Measurement of limb edema
☐ Insertion of a urinary catheter
☐ Nurse’s actions on admission of a pregnant/parturient/postpartum woman with bleeding
☐ Pulse oximetry
☐ Care of a patient with bronchial asthma
☐ Care of a patient with non-infectious gastroenteritis and colitis (age 0–18 years)
☐ Antibiotic sensitivity testing
☐ Nursing visit and nursing care in COVID-19 in children and adolescents

- ☐ Nursing visit and nursing care at first contact in a polyclinic/filter with signs of acute respiratory viral infection, including COVID-19
- ☐ Feeding a severely ill patient via a nasogastric tube
- ☐ Treatment of pressure ulcers
- ☐ Oxygen administration via nasal cannula or oxygen mask (oxygen therapy)

16. Is there a need for nursing documentation?

- ☐ Yes
- ☐ No

17. Do you receive additional payment for an expanded scope of services?

- ☐ Yes
- ☐ No

18. Do you read foreign scientific publications on nursing care and the organization of nursing services?

- ☐ Yes
- ☐ No

19. What is evidence-based nursing practice? (free text) ________

20. What is a nursing diagnosis?

- ☐ A diagnosis according to ICD-10
- ☐ A diagnosis according to the international nursing classification Clinical Care Classification (CCC)

21. Do you know that the international nursing classification Clinical Care Classification (CCC) has been adapted in Kazakhstan?

- ☐ Yes
- ☐ No

22. Do you know that tariffs for nursing care have been introduced in Kazakhstan, according to the Order of the Ministry of Health of the Republic of Kazakhstan of 23 November 2020 No. MoH RK-199/2020 "On approval of the rules for the provision of nursing care" (paragraph 6, Article 127 of the Code of the Republic of Kazakhstan of 7 July 2020 "On the Health of the People and the Healthcare System")?

- ☐ Yes
- ☐ No

23. Do you know that clinical nursing guidelines have been developed and approved in Kazakhstan?

- ☐ Yes
- ☐ No

24. Which clinical nursing guidelines (CNGs) do you use in your work? (select all that apply)

- ☐ Prevention and treatment of pressure ulcers
- ☐ Patient- and family-centered care
- ☐ Anti-smoking interventions in the everyday practice of a nurse
- ☐ Care of an adult patient with asthma: supporting asthma control
- ☐ Chronic heart failure: clinical guideline for nurses (primary health care and inpatient)
- ☐ Adapted clinical nursing guideline for the management of children with cerebral palsy
- ☐ Rehabilitation interventions in stroke. Clinical nursing guideline

- ☐ Prevention of falls and reduction of fall-related injuries. Adapted clinical nursing guideline
- ☐ Management of chronic pain. Adapted clinical nursing guideline
- ☐ Type 2 diabetes mellitus in adults. Adapted clinical nursing guideline
- ☐ Cardiovascular risk assessment and prevention
- ☐ Screening, assessment and care of adult patients with malignant neoplasm with accompanying physiological distress and subsequent depression
- ☐ Adapted clinical nursing guideline for work with persons using potent (psychoactive) substances
- ☐ Ensuring continuity of care
- ☐ Cardiac rehabilitation

25. Has your approach to nursing care changed after completing an applied/academic bachelor's degree?

- ☐ Yes
- ☐ No

26. Do you consider the introduction of the position of nursing service coordinator at the regional health department correct?

- ☐ Yes
- ☐ No

27. Which standard operating procedures (SOPs) do you use in your work? (select all that apply)

- ☐ SOP "Hygienic foot care in patients with diabetes mellitus"
- ☐ SOP "Blood pressure measurement in patients"
- ☐ SOP "Body weight measurement in a patient with CHF"
- ☐ SOP "Assessment of foot vibration sensitivity in patients with diabetes mellitus"
- ☐ SOP "Assessment of foot vessels in patients with diabetes mellitus"
- ☐ SOP "Assessment of foot tactile sensitivity in patients with diabetes mellitus"
- ☐ SOP "Assessment of foot temperature sensitivity in patients with diabetes mellitus"
- ☐ SOP "Teaching a patient with CHF self-care"
- ☐ SOP "Determination of daily diuresis and water balance in CHF"
- ☐ SOP "Foot examination in patients with diabetes mellitus"
- ☐ SOP "Prevention of injuries and falls"
- ☐ SOP "Recognition of white-coat hypertension by an advanced practice nurse"
- ☐ SOP "Algorithm of actions of a medical registrar when communicating with a patient"
- ☐ SOP "Restorative breathing exercises"
- ☐ SOP "Restoration of motor function in patients with cerebral stroke"
- ☐ SOP "Pulse assessment at the radial artery"
- ☐ SOP "Cognitive rehabilitation of post-stroke patients"
- ☐ SOP "Counseling of a patient with CHF in need of palliative care"
- ☐ SOP "Monitoring the condition of a patient with end-stage CHF"
- ☐ SOP "Intensive care unit: nurse's tactics in severe preeclampsia and eclampsia"
- ☐ SOP "Providing individual care for a patient with end-stage CHF"
- ☐ SOP "Assessment of hand function using the MACS in children with cerebral palsy"
- ☐ SOP "Assessment using the Communication Function Classification System (CFCS) in children with cerebral palsy"
- ☐ SOP "Assessment using the Eating and Drinking Ability Classification System (EDACS) in children with cerebral palsy"

☐ SOP “Assessment of a patient’s level of physical activity”
☐ SOP “Rules of interaction with an aggressive or stressed patient”
☐ SOP “Computerized spirography”
☐ SOP “Nurse’s tactics on admission of a pregnant/parturient/postpartum woman with bleeding”
☐ SOP “Physiotherapeutic measures in stroke”
☐ SOP “Mirror therapy for patients with cerebral stroke”

The online form was administered via Google Forms (link provided in the original questionnaire).
