# Supplementary material for: Reported Use of Clinical Guidelines and Nursing Documentation Checklists Among Nurses in Kazakhstan: A Cross-Sectional Online Survey
Source: Healthcare (Basel). 2026 Nov 5;14(21):3780. doi: 10.3390/healthcare14213780 (PMC13763343; doi:10.3390/healthcare14213780)
Supplement: Supplementary file 1 [file healthcare-14-03780-s001.zip › supp m/File_S2_Questionnaire_original.pdf]

АНКЕТИРОВАНИЕ / САУАЛНАМА

Уважаемый участник опроса! Данное анкетирование проводится в рамках исследования по совершенствованию сестринской службы в Республике Казахстан путем разработки и внедрения сестринской документации в организациях практического здравоохранения. Анкета является анонимной, и ее результаты будут использоваться только в обобщенном виде. Заранее благодарим за участие!

Құрметті сауалнамаға қатысушы! Бұл сауалнама практикалық денсаулық сақтау ұйымдарында мейірбикелік құжаттаманы жасау және енгізу арқылы Қазақстан Республикасының мейірбикелік қызметті дамытуды зерттеу аясында жүргізіледі. Сауалнама аноним түрде жүргізіледі және оның нәтижелері тек жалпылама қолданылады. Алдын-ала алғыс айтамыз!

1. Возраст /Жасыңыз

2. Укажите Ваш пол / Жынысыңызды көрсетіңіз
   - ☐ Мужской / Ер
   - ☐ Женский / Әйел

3. Образование/ Біліміңіз
   - ☐ Техническое и профессиональное по специальности «Сестринское дело»/ «Мейіргер ісі»мамандығы бойынша техникалық және кәсіптік/
   - ☐ Прикладной бакалавр по специальности «Сестринское дело»/ «Мейіргер ісі» бойынша қолданбалы бакалавр
   - ☐ Академический бакалавр по специальности «Сестринское дело»/ «Мейіргер ісі» мамандығы бойынша академиялық бакалавр
   - ☐ Магистр по специальности «Сестринское дело»/ «Мейіргер ісі» мамандығы бойынша магистр
   - ☐ PhD по специальности «Сестринское дело»/ «Мейіргер ісі» бойынша PhD

4. Должность/ Қызметіңіз
   - ☐ Медицинская сестра общей практики/ Жалпы тәжірибелік мейіргер
   - ☐ Медицинская сестра расширенной практики/ Кеңейтілген тәжірибелік
   - ☐ Медицинская сестра специализированная/Мамандандырылған мейіргер
   - ☐ Старшая медицинская сестра/ Аға мейіргер
   - ☐ Главная медицинская сестра/ Бас мейіргер
   - ☐ Заместитель директора по сестринскому делу/ Мейіргер ісі бойынша директор орынбасары
   - ☐ Директор центра по сестринскому уходу/ Мейіргерлік күтім орталығының директоры

5. Стаж работы: / Еңбек өтілі: ________________

6. Квалификационная категория: /Біліктілік санатыңыз:
   - ☐ Высшая/ Жоғары
   - ☐ Первая/ Бірінші
   - ☐ Вторая/ Екінші
   - ☐ Нет категории/ Санат жоқ

7. В какой медицинской организации Вы работаете? /Сіз қандай медициналық мекемеде қызмет атқарасыз?*________________

8. Является ли Ваша организация пилотной по развитию сестринской службы ?/ Сіз қызмет атқаратын мекеме мейірбикелік қызметті дамыту бойынша пилотты ма ?
   - ☐ Да/ Ия
   - ☐ Нет/ Жоқ

8.1 ***Источник: Приказ от 4.07.2018 №419 "О реализации пилотного проекта по внедрению новой модели сестринской службы в организациях здравоохранения".***
Дереккөз: 4.07.2018 жылғы №419 Бұйрық "Денсаулық сақтау ұйымдарында мейіргер қызметінің жаңа моделін енгізу бойынша пилоттық жобаны іске асыру туралы".

- ☐ http://www.rcrz.kz/docs/sestrdel/%D0%9F%D1%80%D0%B8%D0%BA%D0%B0%D0%B7%20%D0%A1%D0%94%20%D1%80%D1%83%D1%81%D1%81%D0%BA.PDF

9. Пропишите свой функционал работы / Қызметіңізге не кіреді жазсаңыз ____

10. Знаете ли Вы, что в Казахстане внедрена должность "заместитель руководителя по сестринскому делу"? / Қазақстанда «мейіргер ісі бойынша жетекші орынбасары» лауазымы енгенін білесіз бе?
   - ☐ Да/ Ия
   - ☐ Нет/ Жоқ

11. Знаете ли Вы, что в Казахстане внедрена должность «медсестра расширенной практики»? / Қазақстанда «кеңейтілген тәжірибелік мейіргер» лауазымы енгізілгені жөнінде естідіңіз бе?
   - ☐ Да/ Ия
   - ☐ Нет/ Жоқ

12. Ведете ли Вы, сестринскую документацию? / Сіз мейіргер құжаттарын толтырасыз ба?
   - ☐ Да / Ия
   - ☐ Нет/ Жоқ

13. Ведете ли Вы, сестринский прием пациента?/ Сіз науқасқа мейіргерлік қабылдау жүргізесіз бе?
   - ☐ Да/ Ия
   - ☐ Нет/ Жоқ

14. Если да, какой сбор данных ведете при первичном сестринском приеме? Егер «иә» болса, біріншілк мейіргерлік қабылдауда қандай ақпаратты жинайсыз? ____________________

15. Какую сестринскую документацию (чек-листы) Вы, используете при первичном сестринском приеме? / Біріншілік мейірбикелік қабылдауда Сіз қандай мейіргерлік құжаттарды (чек-листтерді) қолданасыз?
   - ☐ Контроль пациента с острым нарушением мозгового кровообращения / Ми қанайналымының жіті бұзылулары бар пациентті бақылау
   - ☐ Контроль пациента с глаукомой / Глаукомасы бар науқасты бақылау
   - ☐ Помощь при аллергической реакции / Аллергиялық реакция кезінде көмек
   - ☐ Оказание помощи пациенту с переломами/травмами / Сынық/жарақаттанған емделушіге көмек көрсету
   - ☐ Профилактика травм и падений / Жарақаттар мен құлаудың алдын алу
   - ☐ Сахарный диабет у взрослых / Ересектердегі қант диабеті
   - ☐ Сахарный диабет у детей и подростков / Балалар мен жасөспірімдердегі қант диабеті
   - ☐ Уход за венозным катетером / Веноздық катетерге күтім жасау
   - ☐ Обучение пациента с ХСН по уходу за собой / СЖЖ бар науқасқа өзін күтуді үйрету
   - ☐ Уход после трахеостомии / Трахеостомиядан кейінгі күтім
   - ☐ Распознавание Гипертензии Белого Халата / Ақ Халат Гипертензиясын анықтау
   - ☐ Определение суточного диуреза и водного баланса при хронической сердечной недостаточности/ Созылмалы жүрек жеткіліксіздігінде тәуліктік диурез және су балансын анықтау
   - ☐ Действия медицинской сестры при выявлении пациента с инфекционным заболеванием в отделении / Бөлімшеде инфекциялық ауруы бар науқас анықталғандағы мейірбикенің жұмысы
   - ☐ Оценка возможности использования рук по Системе классификации MACS (The Manual Ability Classification System) у детей с церебральным параличом / Цереборалды параличі бар балаларда MACS классификациясы жүйесі бойынша қолды жұмыс істете алу мүмкіндігін бағалау
   - ☐ Введение лекарственных средств с помощью перфузора / Перфузор көмегімен дәрілік заттарды енгізу
   - ☐ Проведение дуоденального зондирования / Дуоденалды зонд қою
   - ☐ Установка желудочного зонда у новорожденных и недоношенных детей / Жаңа туған және шала туған сәбилерде асқазан зондын орнату
   - ☐ Измерение отеков конечностей / Аяқ-қол ісінуін өлшеу
   - ☐ Постановка мочевого катетера / Зәр шығару катетерін қою
   - ☐ Тактика медицинской сестры при поступлении беременной/роженицы/родильницы с кровотечением / Қан кету орын алған жүкті/босанған әйелді қабылдау кезіндегі мейіргер тактикасы
   - ☐ Проведение пульсоксиметрии / Пульсоксиметрия
   - ☐ Уход за пациентом с бронхиальной астмой / Бронх демікпесі бар науқастың күтімі
   - ☐ Уход за пациентом с неинфекционным гастроэнтеритом и колитом (возраст: от 0 до 18 лет) / Инфекциялық емес гастроэнтериті және колиті бар науқастың күтімі (0-ден 18-ге дейінгі жас);
   - ☐ Проведение пробы на чувствительность к антибиотикам / Антибиотикке сезімталдық сынамасын жүргізу;

- ☐ Сестринский прием и сестринский уход при COVID -19 у детей и подростков / Балалар мен жасөспірімдерде COVID -19 кезінде мейіргерлік қабылдау және мейірбикелік күтім
- ☐ Сестринский прием и сестринский уход при первичном обращении пациента в поликлинику/фильтр с признаками ОРВИ, в том числе COVID -19 / Науқас поликлиникаға/фильтрға ЖРВИ, оның ішінде COVID -19 бойынша шағымданғанда мейіргерлік қабылдау және мейіргерлік күтім
- ☐ Кормление тяжелобольного пациента через назогастральный зонд / Ауыр жағдайдағы науқасты назогастралды зондпен тамақтандыру
- ☐ Обработка пролежней / Ойық жараларды өңдеу
- ☐ Подача кислорода с помощью назальной канюли или кислородной маски (оксигенотерапия) / Назалды канюля және оттегі маскасы (оксигенотерапия) көмегімен науқасқа оттегін беру

16. Есть ли потребность в сестринской документации? / Мейіргерлік құжаттамаға қажеттілік бар ма?
    - ☐ Да/ Ия
    - ☐ Нет/ Жоқ

17. Получаете ли Вы, доплату за расширенный объем оказанных услуг? / Көрсетілген қосымша қызметіңіз үшін үстемақы аласыз ба?
    - ☐ Да / Иә
    - ☐ Нет/ Жоқ

18. Читаете ли Вы, зарубежные научные публикации по оказанию сестринского ухода и организации сестринской помощи? / Мейіргелік күтім және мейірбикелік көмекті ұйымдастыру жөнінде шетелдік ғылыми мақалаларды оқисыз ба?
    - ☐ Да / Ия
    - ☐ Нет/ Жоқ

19. Что такое доказательная сестринская практика? / Дәлелді мейірбикелік тәжірибе деген не?

20. Что такое сестринский диагноз? / Мейіргерлік диагноз деген не?
    - ☐ Диагноз по МКБ 10 / МКБ 10 бойынша диагноз
    - ☐ Диагноз по международного классификатора сестринской практики Clinical Care Classification(CCC)/ Мейіргерлік тәжірибенің халықаралық классификаторы бойынша (Clinical Care Classification(CCC)) диагноз

21. Знаете ли Вы, что в Казахстане адаптирован международный классификатор сестринской практики Clinical Care Classification(CCC)? / Мейіргерлік тәжірибенің халықаралық классификаторы Clinical Care Classification(CCC) Қазақстанға сай бейімделгенін білесіз бе?
    - ☐ Да / Ия
    - ☐ Нет / Жоқ

22. Знаете ли Вы, что в Казахстане внедрены тарифы по сестринскому уходу, согласно приказу: МЗ РК от 23 ноября 2020 года № ҚР ДСМ Приказ М-199/2020 Об утверждении правил оказания сестринского ухода (пункт 6 Статья 127 Кодекса РК от 7 июля 2020 года «О здоровье народа и системе здравоохранения»). / Қазақстанда ҚР ДСМ 23 қараша 2020 жыл № М-199/2020 бұйрығымен (Мейірбикелік күтім ережелерін бекіту жөнінде) («Халық денсаулығы және денсаулық сақтау жүйесі жөнінде» 2020 ж. 7 шілдедегі ҚР Кодексінің 6-пункті, 127 статья) . мейірбикелік күтім бойынша тарифтер енгізілгенін білесіз бе?
    - ☐ Да / Ия
    - ☐ Нет / Жоқ

23. Знаете ли Вы, что в Казахстане разработаны и утверждены клинические сестринские руководства?/ Қазақстанда клиникалық мейіргерлік жетекшілктер әзірленіп, бекітілгенін білесіз бе?
    - ☐ Да / Ия
    - ☐ Нет / Жоқ

24. Отметьте, какие клинические сестринские руководства (КСР) Вы используете в работе? / Сіз қызметте қандай клиникалық мейіргерлік жетекшіліктерді қолданасыз (КМЖ)?
    - ☐ Профилактика и лечение пролежней/ Ойық жаралардың алдын алу және емдеу
    - ☐ Уход, ориентированный на пациента и его семью/ Науқасқа және оның отбасына бағытталған күтім
    - ☐ Применение вмешательств против табакокурения в повседневной практике медицинской сестры/ Мейіргерлік күнделікті тәжірибесінде темекі шегуге қарсы араласулар
    - ☐ Уход за взрослым пациентом с астмой: содействие контролю над астмой/ Астмасы бар ересек науқастың күтімі: астманы бақылауға көмектесу

- ☐ Хроническая сердечная недостаточность: клиническое руководство для медицинских сестер по ведению больных (ПСМП и стационар)/ Созылмалы жүрек жеткіліксіздігі: науқастарға қарау бойынша мейіргерлік клиникалық жетекшілік (амбулатория және стационар)
- ☐ Адаптированное клиническое сестринское руководство по ведению детей с церебральным параличом/ / Церебралды параличі бар балаларға бейімделген клиникалық мейірбикелік жетекшілік
- ☐ Реабилитационные мероприятия при инсульте. Клиническое сестринское руководство/ Инсульт кезіндегі реабилитациялық іс-шаралар. КМЖ.
- ☐ Предотвращение падений и снижение травматизма при падениях. Адаптированное клиническое сестринское руководство/ Құлаудың алдын алу және құлау кезіндегі травматизмді азайту. Бейімделген КМЖ
- ☐ Управление хронической болью. Адаптированное клиническое сестринское руководство/Созылмалы ауырсынуды басқару. Бейімделген клиникалық мейіргерлік нұсқаулық
- ☐ Сахарный диабет 2 типа у взрослых. Адаптированное клиническое сестринское руководство/Ересектердегі 2 типті қант диабеті. Бейімделген клиникалық мейіргерлік нұсқаулық
- ☐ Оценка риска и профилактика сердечно-сосудистых заболеваний/ Жүрек-қантамыр ауруларының қаупін бағалау және алдын алу
- ☐ Скрининг, оценка и уход за взрослыми пациентами, страдающими злокачественным новообразованием с сопутствующим физиологичным дистрессом и последующей депрессией/ Физиологиялық дистресс және депрессиямен жүретін қатерлі ісігі бар ересек науқастарға скрининг жасау, оны бағалау және күтім жасау
- ☐ Адаптированное клиническое сестринское руководство по работе с категориями лиц, употребляющими сильнодействующие вещества/ Қатты әсер ететін ДЗ қолданатын адамдарға арналған бейімделген КМЖ
- ☐ Обеспечение непрерывного ухода/ Үздіксіз күтім жасау
- ☐ Сердечная реабилитация/ Жүрек реабилитациясы

25. Изменился ли у Вас подход к оказанию сестринского ухода после окончания прикладного / академического бакалавриата: / Қолданбалы / академиялық бакалавриатты аяқтаған соң мейірбикелік күтім жасаудағы әрекетіңіз өзгреді ме?
    - ☐ Да/ Ия
    - ☐ Нет/ Жоқ

26. Считаете ли Вы правильным внедрение должности координатора по сестринской службе при управлении здравоохранения области? / Мейіргелік қызмет бойынша координатор лауазымын енгізуді дұрыс деп санайсыз ба (денсаулық сақтау саласы басқарса)?
    - ☐ Да/ Ия
    - ☐ Нет/ Жоқ

27. Отметьте, какие стандарты операционных процедур (СОП) Вы используете в работе? / Сіз қызметіңізде операциялық процедуралардың қандай стандарттарын пайдаланасыз? (ОПС)

    - ☐ Стандарт операционных процедур "Гигиеническая обработка ног у больных сахарным диабетом";/ ОПС "Қант диабеті бар науқастарда аяқты гигиеналық өңдеу ";
    - ☐ Стандарт операционных процедур "Измерение артериального давления у пациентов";/ ОПС "Науқастарда артериальды қысым өлшеу ";
    - ☐ Стандарт операционных процедур "Измерение массы тела пациента при ХСН";/ ОПС "СЖЖ кезінде науқастың дене сламағын өлшеу";
    - ☐ Стандарт операционных процедур "Исследование вибрационной чувствительности стоп у больных сахарным диабетом";/ ОПС "Қант диабеті бар науқастарда табанның вибрациялық сезімталдығын зерттеу";
    - ☐ Стандарт операционных процедур "Исследование сосудов стопы у больных сахарным диабетом";/ ОПС "Қант диабеті бар науқастарда табанның тамырларын зерттеу";/
    - ☐ Стандарт операционных процедур "Исследование тактильной чувствительности стоп у больных сахарным диабетом";/ ОПС «Қант диабеті бар науқастарда табанның тактильді сезімталдығын зерттеу»;
    - ☐ Стандарт операционных процедур "Исследование температурной чувствительности стоп у больных сахарным диабетом";/ ОПС "Қант диабеті бар науқастарда табанның температуралық сезімталдығын зерттеу";\
    - ☐ Стандарт операционных процедур "Обучение пациента с ХСН по уходу за собой";/ ОПС "СЖЖ бар науқасқа өзін күтуді үйрету";
    - ☐ Стандарт операционных процедур "Определение суточного диуреза и водного баланса при ХСН";/ ОПС "СЖЖ кезінде тәуліктік диурез және су балансын анықтау";

- ☐ Стандарт операционных процедур "Осмотр стоп у больных сахарным диабетом";/ ОПС "Қант диабеті бар науқастарда табанды қарау";
- ☐ Стандарт операционных процедур "Профилактика травм и падений";/ ОПС "Құлау және травманың алдын алу ";
- ☐ Стандарт операционных процедур "Распознавание Гипертензии Белого Халата медсестрой расширенной практики";/ ОПС "Кеңейтілген тәжірибелі мейірбикенің ақ халат гипертензияын анықтауы";
- ☐ Стандарт операционных процедур "Алгоритм действий медицинского регистратора при общении с пациентом";/ ОПС "Медициналық тіркеушінің науқаспен сөйлесудегі әрекет алгоритмі";
- ☐ Стандарт операционных процедур "Восстановительная дыхательная гимнастика";/ ОПС "Қалпына келтіретін тыныс гимнастикасы";
- ☐ Стандарт операционных процедур "Восстановление моторики у пациентов с церебральным инсультом";/ ОПС "Церебралды инсульт алған науқастарда моториканы қалыпқа келтіру";
- ☐ Стандарт операционных процедур "Исследование пульса у пациента на лучевой артерии";/ ОПС "Науқастың кәріжілік артериясынан пульсты анықтау";
- ☐ Стандарт операционных процедур "Когнитивная реабилитация постинсультных больных";/ ОПС "Инсульттан кейінгі науқастардың когнитивті реабилитациясы";
- ☐ Стандарт операционных процедур "Консультирование пациента с ХСН, нуждающегося /в паллиативной помощи";/ ОПС "Паллиативті көмекке мұқтаж СЖЖ бар науқасқа консультация беру";
- ☐ Стандарт операционных процедур "Мониторинг состояния пациента с ХСН в терминальной стадии";/ ОПС "Терминалды деңгейдегі СЖЖ бар науқастың жағдайына мониторинг";
- ☐ Стандарт операционных процедур "ОАРИТ тактика медицинской сестры при преэклампсии тяжелой степени и эклампсии";/ ОПС "Ауыр дәрежелі преэклампсия және эклампсия кезіндегі АРИТБ мейірбике тактикасы ";
- ☐ Стандарт операционных процедур "Обеспечение индивидуального ухода за пациентом с терминальной ХСН";/ ОПС "Терминалды СЖЖ бар науқастың индивидуалды күтімін жасау ";
- ☐ Стандарт операционных процедур "Оценка возможности использования рук по Системе классификации MACS у детей с церебральным параличом";/ ОПС "Цереборалды параличі бар балаларда MACS классификациясы жүйесі бойынша қолды жұмыс істете алу мүмкіндігін анықтау ";
- ☐ Стандарт операционных процедур "Оценка по Системе классификации коммуникативной функции (CFCS) у детей с церебральным параличом";/ ОПС " Церебралды параличі бар балаларды коммуникативті функция классификациясының жүйесі (CFCS)бойынша бағалау ";
- ☐ Стандарт операционных процедур "Оценка по системе классификации способности принимать пищу и пить (EDACS) у детей с церебральным параличом";/ ОПС " Церебралды параличі бар балаларды тамақтана алу және сусын іше алу қабілеті бойынша классификациялау (EDACS) жүйесімен бағалау";
- ☐ Стандарт операционных процедур "Оценка уровня физической активности пациента"; / ОПС "Науқастың физикалық белсенділік деңгейін анықтау";
- ☐ Стандарт операционных процедур "Правила взаимодействия с агрессивным или испытывающим стресс пациентом";/ ОПС "Агрессивті немесе стресстегі науқаспен арақатынас ережелері";
- ☐ Стандарт операционных процедур "Проведение компьютерной спирографии"; / ОПС "Компьютерлік спирографияны жүргізу";
- ☐ Стандарт операционных процедур "Тактика медицинской сестры при поступлении беременной роженицы родильницы с кровотечением";/ "Жүкті босанған әйелді қан кетумен қабылдау кезіндегі мейірбикенің тактикасы"операциялық процедуралардың стандарты;
- ☐ Стандарт операционных процедур "Физиотерапевтические мероприятия при инсульте"; / ОПС "Инсульт кезіндегі физиотерапиялық іс-шаралар";
- ☐ Стандарты операционных процедур "Зеркальная терапия пациентам с церебральным инсультом";/ ОПС "Церебралды инсульт алған науқастарда «айналы» терапия";

Ссылка: https://forms.gle/kxvYG5Jv2ULpTiGh8
