# Supplementary material for: Reported Use of Clinical Guidelines and Nursing Documentation Checklists Among Nurses in Kazakhstan: A Cross-Sectional Online Survey
Source: Healthcare (Basel). 2026 Nov 5;14(21):3780. doi: 10.3390/healthcare14213780 (PMC13763343; doi:10.3390/healthcare14213780)
Supplement: Supplementary file 1 [file healthcare-14-03780-s001.zip › supp m/File_S3_Invitation_letter_redacted.pdf]

«СЕМЕЙ МЕДИЦИНА УНИВЕРСИТЕТІ» КОММЕРЦИЯЛЫҚ ЕМЕС АКЦИОНЕРЛІК ҚОҒАМЫ

SMU
1953 SEMEY MEDICAL UNIVERSITY

НЕКОММЕРЧЕСКОЕ АКЦИОНЕРНОЕ ОБЩЕСТВО «МЕДИЦИНСКИЙ УНИВЕРСИТЕТ СЕМЕЙ»

071400, Қазақстан Республикасы,
Семей қаласы, Абай көшесі, 103
Телефон/факс:8(7222)52-22-51, 56-97-55
E-mail: smu@nao-mus.kz
Веб-сайт: www.semeymedicaluniversity.kz

071400, Республика Казахстан,
город Семей, улица Абая,103
телефон/факс: 8(7222)52-22-51, 56-97-55
E-mail: smu@nao-mus.kz
Веб-сайт: www.semeymedicaluniversity.kz

№ 06-02/6372
21.12.2021

Руководителям медицинских организаций ПМСП Республики Казахстан

НАО «Медицинский университет Семей» выражает свое почтение организациям практического здравоохранения и просит Вас оказать содействие в проведении онлайн-опроса в рамках научного исследования докторанта второго года обучения по специальности «Общественное здравоохранение» Сыдықовой Бибінұр Қабдығалиқызы на тему: **«Совершенствование сестринской службы в Республике Казахстан путем разработки и внедрения сестринской документации в организациях практического здравоохранения».** К участию в исследовании приглашаются медицинские сёстры с уровнем технического и профессионального образования (ТиПо) и прикладного/академического бакалавриата в области сестринского дела, которым предлагается ответить на вопросы специализированного опросника по нижеследующей ссылке:

Ссылка: https://forms.gle/kxvYG5Jv2ULpTiGh8

Анкетирование является конфиденциальным и анонимным. Данные будут использоваться в обобщенном виде. При необходимости исследователи могут предоставить Вам отчет о результатах анкетирования по Вашей организации, без права публикации (контакт для связи- Сыдықова Б.Қ., +7[illegible]

Благодарим за содействие и надеемся на дальнейшее плодотворное сотрудничество.

Заместитель Председателя Правления по научно-клинической работе НАО «МУС» **Танатарова Г.Н**

Исп: Сыдықова Б.Қ
Тел[illegible]

003133
