# Supplementary material for: Reported Use of Clinical Guidelines and Nursing Documentation Checklists Among Nurses in Kazakhstan: A Cross-Sectional Online Survey
Source: Healthcare (Basel). 2026 Nov 5;14(21):3780. doi: 10.3390/healthcare14213780 (PMC13763343; doi:10.3390/healthcare14213780)
Supplement: Supplementary file 1 [file healthcare-14-03780-s001.zip › supp m/Supplementary_Table_S1.pdf]

Supplementary Table S1. Reported use of the 16 checklists among respondents who conduct an initial nursing visit (item 13 = “Yes”; n = 1,715)

Whole-sample counts are those in Tables 3–4. Counts among respondents with item 13 = “Yes” were calculated from the raw response data (Google Forms export) after exclusion of the 13 invalid forms (N = 2,263) and may differ from the whole-sample counts by a few respondents.

Checklist	Whole sample (N = 2,263), n (%)	Among item 13 = “Yes” (n = 1,715), n (% [95% CI])
Allergic reaction management	912 (40.3%)	707 (41.2% [38.9–43.6])
Injury and fall prevention	781 (34.5%)	589 (34.3% [32.1–36.6])
Infectious disease on the ward	683 (30.2%)	487 (28.4% [26.3–30.6])
Acute stroke monitoring	622 (27.5%)	519 (30.3% [28.1–32.5])
Diabetes mellitus, adults	525 (23.2%)	431 (25.1% [23.1–27.2])
Fractures/injuries	518 (22.9%)	417 (24.3% [22.3–26.4])
Venous catheter care	441 (19.5%)	362 (21.1% [19.2–23.1])
CHF: patient education	440 (19.4%)	374 (21.8% [19.9–23.8])
CHF: diuresis/water balance	330 (14.6%)	261 (15.2% [13.6–17.0])
Perfusor administration	329 (14.5%)	241 (14.1% [12.5–15.8])
Glaucoma monitoring	322 (14.2%)	279 (16.3% [14.6–18.1])
Diabetes mellitus, children	308 (13.6%)	263 (15.3% [13.7–17.1])
White-coat hypertension	286 (12.6%)	247 (14.4% [12.8–16.1])
Tracheostomy care	248 (11.0%)	193 (11.3% [9.8–12.8])
Neonatal gastric tube	202 (8.9%)	145 (8.5% [7.2–9.9])
Duodenal probing	194 (8.6%)	157 (9.2% [7.9–10.6])
